# Establishing a jugular-femoral venous route for recanalization of complicated inferior vena cava in Budd–Chiari Syndrome after transfemoral access failure

**DOI:** 10.1038/s41598-022-07935-5

**Published:** 2022-03-10

**Authors:** Yonghua Bi, Zhengyang Wu, Mengfei Yi, Xinwei Han, Jianzhuang Ren

**Affiliations:** Department of Interventional Radiology, The First Affiliated Hospital of Zhengzhou University, No.1, East Jian She Road, Zhengzhou, 450052 China

**Keywords:** Anatomy, Diseases

## Abstract

Recanalization of inferior vena cava (IVC) with complete obstruction, old thrombosis or long segmental stenosis/obstruction (complicated IVC) via transfemoral access may fail in patients with Budd-Chiari syndrome (BCS). In this study, 34 consecutive patients with BCS underwent recanalization of complicated IVC occlusion via jugular-femoral venous (JFV) route establishment. BCS with complicated IVC was detected by reviewing preoperative color Doppler ultrasonography or computed tomography (CT) venography, and confirmed by intraoperative venography. Clinical data on technical success, complications, and follow-up outcomes were analyzed. Except for one patient received surgical repair of rupture IVC after recanalization, technical success of IVC recanalization was achieved in remaining 33 (97.1%) patients. No perioperative deaths was found. Three complications were observed during recanalization, for a complication rate of 8.8%. Bleeding of the femoral vein was observed in one patient, and two patients showed bleeding of IVC. The IVC lesion diameter and blood flow of the distal IVC increased significantly after the procedure. Twenty-four patients (77.4%) were clinically cured, and four patients (12.9%) showed clinical improvement. The 1-year, 3-year, 5-year primary patency rates were 85.9%, 76.4% and 70.0%, respectively. The 5-year secondary patency rate was 96.8%. There were three deaths during follow up, and the 5-year survival rate was 90.0%. In conclusion, JFV route establishment and angioplasty for complicated IVC is safe and effective for patients with BCS after transfemoral access failure.

## Introduction

In Western countries, Budd-Chiari syndrome (BCS) often involves the hepatic veins^1,2^. By contrast, in Asia occlusion of the inferior vena cava (IVC) is common in BCS^3^. IVC obstruction may be associated with poor standard of living^4^, IVC obstruction may become less common and similar or increasing prevalence of hepatic vein obstruction may be observed in the developing countries with improvement in standards of living^5^. Because it has a high success rate with fewer complications, percutaneous transluminal angioplasty (PTA) should be considered as the first choice for BCS patients with IVC occlusion^6–19^. However, endoluminal recanalization via transfemoral access may fail in complicated IVC, even after recanalization with a J-type Brockenbrough needle^19^ or a steel needle^21^. In our experience, jugular-femoral venous (JFV) route establishment should be performed for angioplasty under this circumstance. To dates, no reports have focused specifically on JFV route establishment for patients with complicated IVC. In this study, we aimed to report the clinical outcomes of JFV route establishment and angioplasty for BCS with complicated IVC.

## Patients and methods

### Clinic data

This retrospective study was approved by the Zhengzhou university ethics committee on human investigation. Written informed consent was obtained from all patients. All methods were performed in accordance with the relevant guidelines and regulations, and the experimental protocol was approved by the Zhengzhou university ethics committee. Between February 2012 and July 2019, 34 consecutive BCS patients who underwent JFV route establishment due to failure in transfemoral access were included in this study. BCS with complicated IVC was detected by reviewing color Doppler ultrasonography (GE Vivid 7) or 64-slice CT venography scanning (Philips Brilliance), and confirmed by intraoperative venography. Patients whose life expectancy life was less than 3 months due to terminal hepatic carcinoma or severe visceral failure, including liver function failure, or severe coagulation dysfunction were excluded from this study. Clinical data on technical success, complications, and follow-up outcomes were collected and analyzed.

### Endoluminal recanalization for complicated IVC

**Figure 1:**
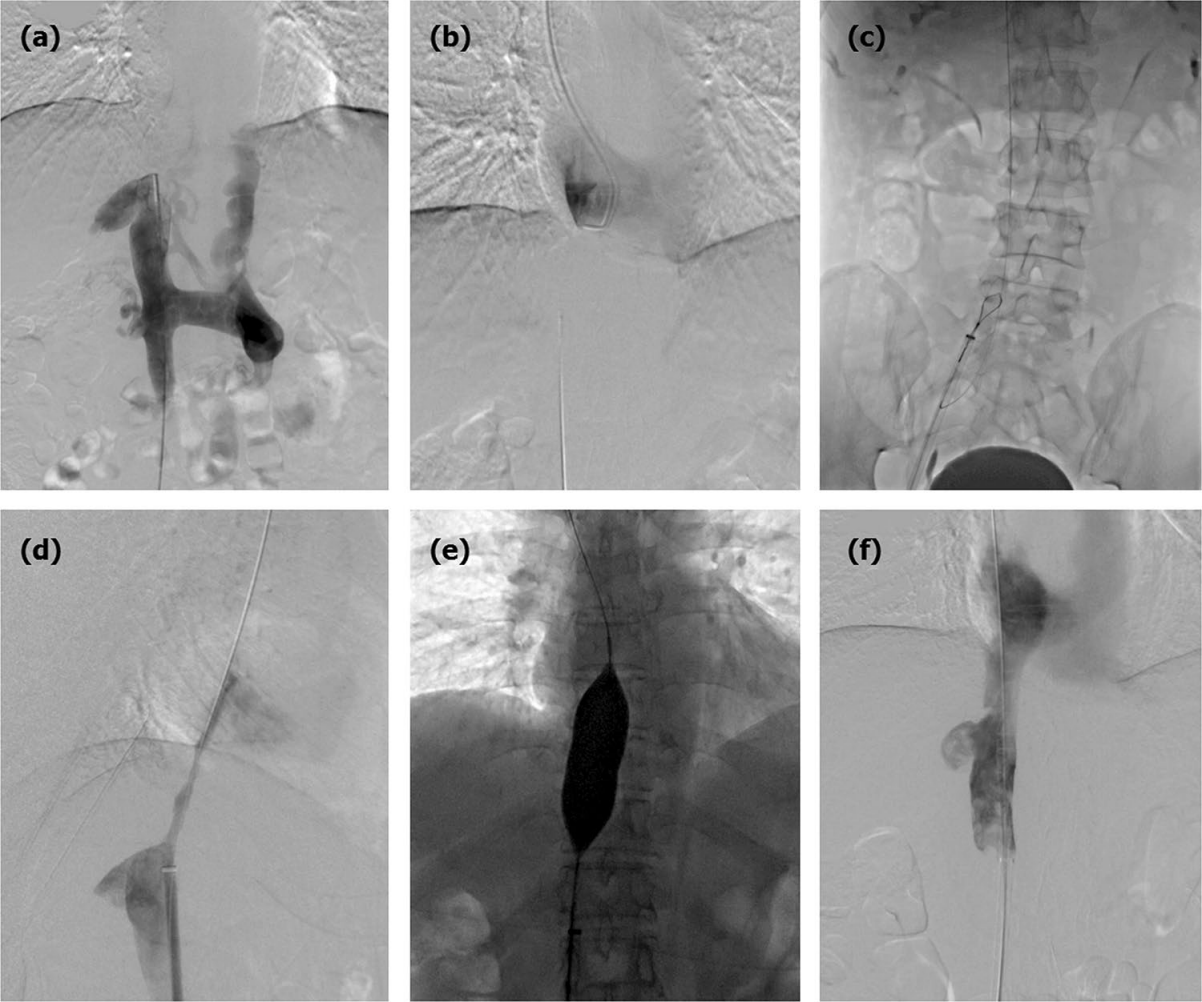
JFV route establishment and IVC recanalization. (**a**) A segmental occlusion was shown in the suprahepatic IVC by venography via femoral access. (**b**) Downward recanalization was performed via jugular access after transfemoral access failed. (**c**) A 0.035-inch stiff guide wire was introduced into the distal IVC, and a gooseneck capture was introduced to pull out the guide wire. (**d**) Venography was performed via a 14 F long sheath after JFV route establishment. (**e**) Angioplasty was performed using a large balloon (30 mm diameter). (**f**) Venography confirmed successful recanalization and widely patent IVC without bleeding.

IVC recanalization was performed via right femoral venous access, and then the right jugular vein was cannulated if the femoral venous access failed. A 5F pigtail catheter was introduced into the IVC for venography. A 0.035-inch guide wire and a 5F headhunter catheter (Cook, Bloomington, IN, USA) were introduced to the IVC for recanalization. If this failed, the rigid end of the guide wire or J-type blunt needle was introduced into the distal part of IVC lesion for sharp recanalization. After withdrawing the guide wire, a 0.035-inch stiff guide wire of 260 cm in length (Amplatz Super Stiff; Boston Scientific, Massachusetts, USA) was introduced into the IVC. A 14F long sheath and gooseneck capture were introduced, and the guide wire was pulled out to establish a JFV route. Predilation was performed via long sheath or small diameter balloon catheter^10,12^. A large diameter balloon catheter (Cook) was inserted for IVC angioplasty via the stiff guide wire (Fig. 1).

### Related interventional procedures

Catheter-directed thrombolysis was performed for patients with fresh thrombosis in IVC^16,17^. A 5-Fr catheter with multiple side holes (Cook, Bloomington, IN, USA) was introduced and positioned in the thrombus segment of IVC to dissolve the fresh thrombus by continuous infusion of urokinase at a rate of 10–20 × 10^5^ U/h for 2 h per day. For patients with massive thrombolysis in IVC, a recoverable stent was inserted to compress the thrombus, and a permanent stent was used if IVC obstruction remained after repeated PTA or thrombolysis^16,17^. Anticoagulation has been recommended for all patients with no contraindications and subcutaneous injections of low-molecular-weight heparin (5100 IU every 12 h) was performed during hospitalization. Then only underwent oral anticoagulant therapy with rivaroxaban after discharge^8,10^.

### Follow-up evaluation and definition

Color Doppler ultrasonography was performed during patient follow-up. Clinical data on technical success, complications, and follow-up outcomes were collected and analyzed. Technical success was defined as a successful establishing of IVC route and IVC recanalization, with no significant residual stenosis, death or serious complications requiring surgical treatment. The JFV route was defined as a guild-wire route connected between the jugular and femoral veins, which helps introduce a balloon or stent for angioplasty of complicated IVC after transfemoral access fails. The relative normal diameter of the proximal IVC and the diameter of the relative normal segment of proximal IVC near the obstruction were measured by ultrasonography. Patients were clinically cured if there were no symptom or signs and the IVC was patent as confirmed by ultrasonography. BCS was classified according to the duration of disease^20^. The clichy prognostic index and new prognostic index were calculated according to the equations reported previously^21,22^.

### Statistical analysis

Data are reported as the mean ± SD, or mean and IQR for abnormal distribution. Student t test and ANOVA were used for analysis. Qualitative data were shown as numbers and percentages. Survival rates were analyzed by using Kaplan–Meier curves. Statistical analyses were carried out using Prism 5.0 (GraphPad Software, Inc., SanDiego, CA).

## Results

### Patient characteristics

A total of 34 BCS patients with complicated IVC (24 men and 10 women; median age 48.5 years, range 22–72 years) were included in this study. Six patients (17.6%) showed a acute BCS, four patients (11.8%) had subacute BCS and 24 patients (70.6%) had chronic BCS. Thirty cases (88.2%) had combined hepatic veins and IVC involvement, and four patients (11.8%) showed isolated IVC involvement. Twelve (35.3%) patients showed membranous obstruction of IVC and 22 cases (64.7%) showed segmental obstruction. Six patients (17.6%) showed IVC thrombosis involvement and three patients (8.8%) had additional deep vein thrombosis. Eighteen patients (53.0%) showed a liver function score of Child Pugh class A, and 10 patients (29.4%) showed a score of Child Pugh class B. The clichy prognostic index and new prognostic index were 4.9 ± 0.9 and 4.0 ± 1.5, respectively (Table 1). Lower extremity edema and abdominal distension were the most common symptoms and signs before IVC recanalization.

**Table 1:** General data of enrolled patients at admission.

Variables	Data
Male, n (%)	24 (70.6%)
Mean age (Range), years	50.4 ± 10.8 (22–72)
**Lesion types**	
IVC and hepatic vein involvement	30 (88.2%)
Isolated IVC involvement	4 (11.8%)
Segmental obstruction	22 (64.7%)
Membranous obstruction	12 (35.3%)
IVC thrombosis involvement	6 (17.6%)
**Duration of symptom, n (%)**	
Less than 1 year	13 (38.2%)
Between 1 and 10 years	10 (29.4%)
More than 10 years	11(32.4%)
**Liver function before procedure, n (%)**	
Child Pugh A	18 (53.0%)
Child Pugh B	10 (29.4%)
Child Pugh C	6 (17.6%)
Clichy prognostic index	4.9 ± 0.9
New prognostic index	4.0 ± 1.5

### Recanalization

The right femoral vein was the routine access vein for IVC recanalization. if this failed, the right internal jugular vein was accessed. IVC recanalization was successfully performed in all 34 patients, 31 of whom needed sharp recanalization. The J-type blunt needle was used for sharp recanalization in 17 (54.8%) patients, and the blunt head of the guide wire was used in 14 (45.2%) patients. Upward recanalization from the femoral vein was the first choice for all patients. Downward recanalization from jugular vein was performed in 28 (90.3%) patients after failure of upward recanalization. Predialation with a long sheath was performed in 12 patients, and 12 patients underwent predialation by means of a small diameter balloon. Large balloon angioplasty was performed for 28 patients with a balloon catheter of 20–30 mm in diameter (Table 2).

**Table 2:** IVC recanalization.

Variables	Data
IVC recanalization	n = 31
J-type blunt needle	17 (54.8%)
Blunt head of guide wire	14 (45.2%)
Upwards recanalization from FV	3 (9.7%)
Downwards recanalization from JV	28 (90.3%)
Diameter of large balloon, mm (n = 28)	25.8 ± 3.0 (range 20–30)
Diameter of small balloon, mm (n = 12)	12.1 ± 3.0 (range 8–16)
Predilation by long sheath	12 (35.3%)
Catheter directed thrombolysis	4 (11.8%)
Retrieval stent placement	2 (5.9%)
Permanent stent placement	3 (8.8%)
**Perioperative complications**	
Bleeding of IVC	2 (5.9%)
Bleeding of femoral vein	1 (2.9%)

*FV* Femoral vein, *JV* Jugular vein, *IVC* Inferior vena cava.

### Related interventional procedures

Four patients underwent catheter-directed thrombolysis for a fresh thrombus in IVC. Two recoverable stents were implanted for BCS with massive IVC thrombus, which were removed after thrombus disappearance was confirmed by ultrasonography. Three permanent vascular stents were used for patients with recurrent IVC obstruction. One patient required transjugular intrahepatic portosystemic stent shunt and none needed hepatic vein stenting.

### Perioperative complications

**Figure 2:**
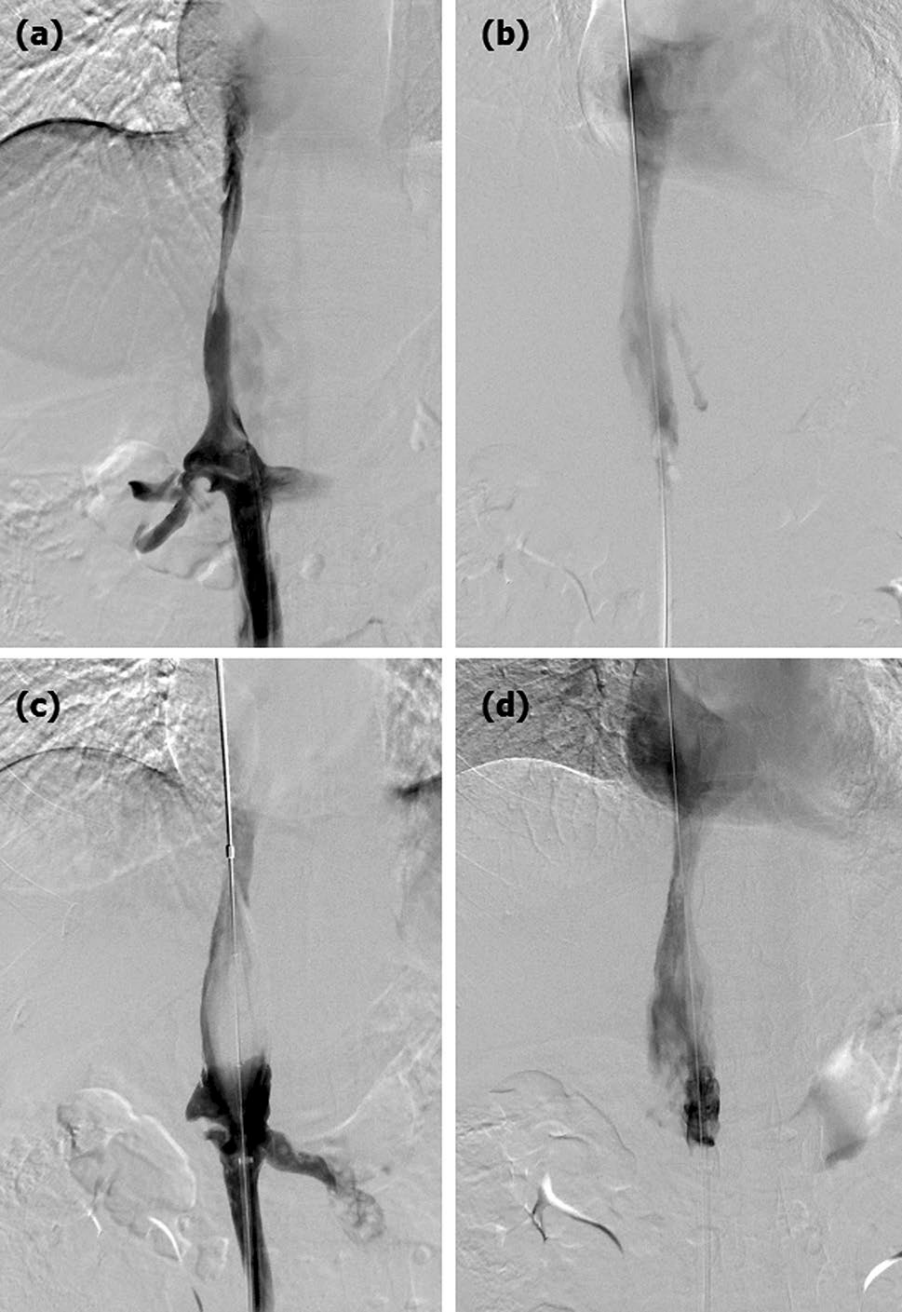
Balloon angioplasty and IVC bleeding. (**a**) Digital subtraction inferior venography showed a severe stenosis of the proximal IVC. (**b**) Immediate bleeding was observed in the dilated segment after balloon angioplasty. (**c**) The 26 mm diameter balloon catheter was introduced and dilated immediately to block bleeding. (**d**) Venography showed the disappearance of bleeding after 15 min without further management.

No perioperative deaths were observed. Three complications were observed during recanalization, for a rate of 8.8%. One patient showed contrast agent outflow in the dilated segment after balloon angioplasty. A 26-mm diameter balloon catheter was introduced and dilated immediately to block bleeding, and no contrast agent was exuded after 15 min (Fig. 2). The other patient showed rupture and bleeding of IVC, and surgical repair was performed after failure of balloon blockage. Bleeding of the femoral vein was found in 1 patient (Table 2).

### Size changes of IVC

**Figure 3:**
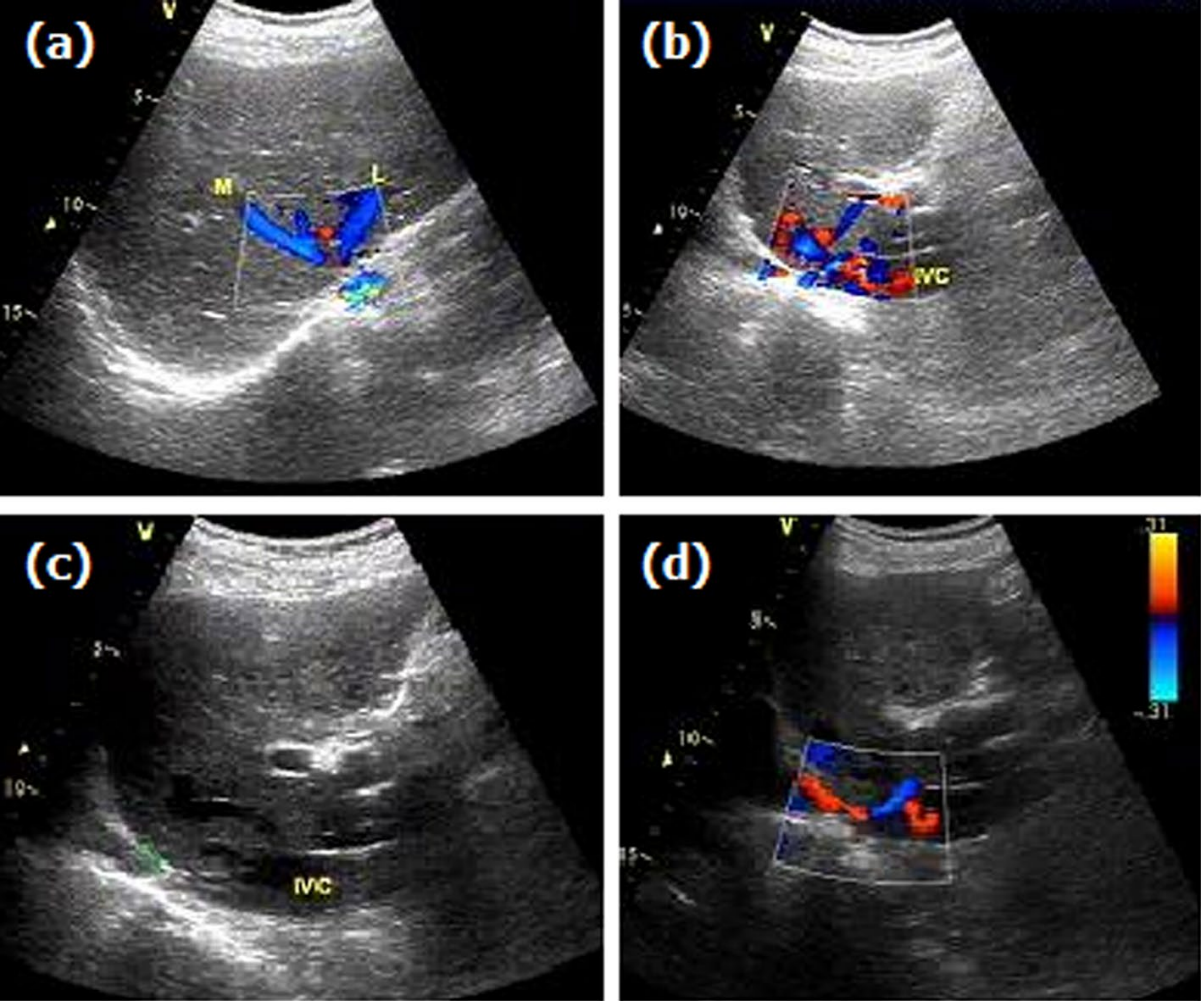
Color Doppler ultrasonography. Segmental occlusion was shown in the proximal IVC (**a**, **b**). Partial patency was shown in the dilated IVC after angioplasty (**c**, **d**).

The median length of occlusive IVC was 18.5 mm before the procedure, which decreased significantly after the procedure (*p* < 0.0001). The mean diameter of the IVC lesion was 3.5 ± 2.5 mm before recanalization, which increased to 7.0 ± 2.0 mm after the procedure (*p* < 0.0001). The relative normal diameter of the proximal IVC did not change significantly within 6 months after the procedure. The diameter of distal IVC decreased significantly after the procedure, which indicated that dilation of the distal IVC had occurred before the procedure due to obstruction (Fig. 3). Blood flow of the distal IVC increased significantly during follow-up (*p* < 0.0001, Table 3).

**Table 3:** Change of IVC before and after procedure.

Variables	Before procedure	After procedure (< 6 months)	After procedure(> months)
Length of occlusive IVC, mm	18.5 (14.0–34.6)	0 (0–0)***	0 (0–0)***
Relative normal diameter of proximal IVC, mm	17.6 ± 4.6	15.1 ± 4.6	13.9 ± 4.3*
Diameter of IVC lesion, mm	3.5 ± 2.5	7.0 ± 2.0***	7.0 ± 2.0***
Diameter of distal IVC, mm	15.1 ± 4.6	11.1 ± 3.0**	11.5 ± 2.9**
Blood speed of IVC (cm/s)	18.5 (0–39.3)	66.0 (43.0–80.0)**	66.0 (47.5–93.5)**

vs Before procedure, *p < 0.05; **p < 0.01; ***p < 0.0001.

### Clinical efficacy evaluation and follow-up

**Figure 4:**
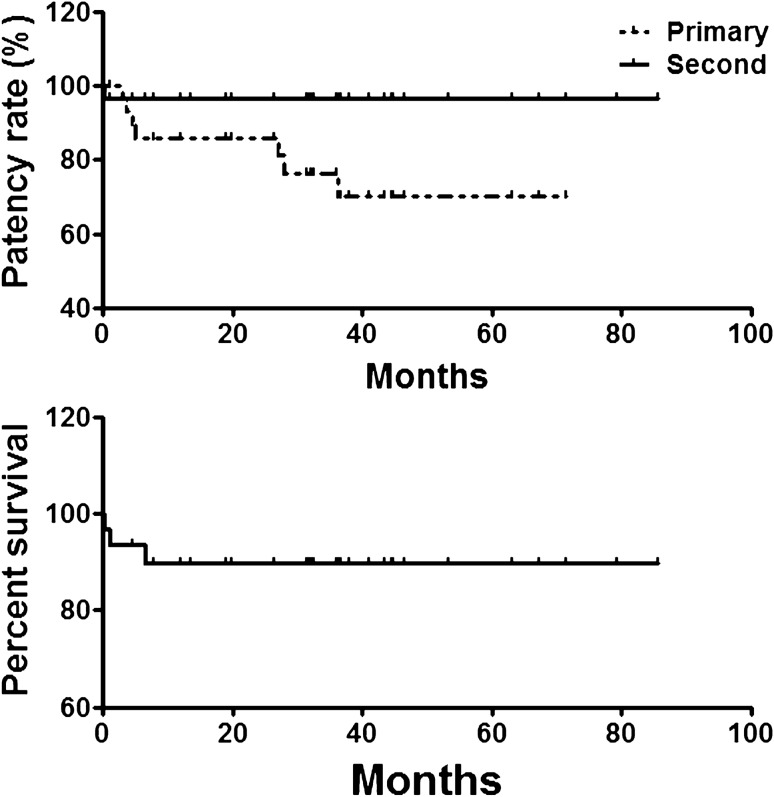
Patency rate and survival rate. The 1-year, 3-year, 5-year primary patency rates were 85.9%, 76.4% and 70.0%, respectively. The 5-year second patency rate was 96.8%. The 5-year survival rate was 90.0%.

Three patients were lost during follow-up, for a loss rate of 8.8%. Twenty-four patients (77.4%) were clinically cured and 4 patients (12.9%) showed clinical improvement. The primary patency rates were 85.9%, 76.4% and 70.0% for 1 year, 3 years, and 5 years, respectively. The 5-year secondary patency rate was 96.8%. There were 3 deaths during follow-up. Two patients died of liver failure after procedure and one patient died of advanced liver cancer about 6 months later. The 5-year survival rate was 90.0% (Fig. 4).

## Discussions

Because it has a high success rate with fewer complication, percutaneous transluminal angioplasty (PTA) should be considered as first choice for BCS patients with membranous IVC occlusion^6,7,9,11,12,15^. However, endoluminal recanalization via femoral venous access may fail in complicated IVC, even after recanalization with a J-type Brockenbrough needle^19^ or a steel needle^23^. Xu et al.^19^ reported IVC recanalization by using a J-type Brockenbrough needle via femoral venous access, which failed in 2 of 103 patients. Ding et al.^23^ reported the use of a steel needle for recanalization of membranous IVC occlusion via femoral venous access. If the transfemoral venous approach fails, JFV route establishment should be performed for angioplasty of complicated IVC with complete obstruction, old thrombosis or long segmental stenosis/obstruction.

For patients with a “v”-shaped of proximal IVC occlusion, or with a small proximal diameter (less than 10 mm), upward recanalization via femoral access not only increases the difficulty of cannulation, but also increases the probability of penetrating the pericardium because the guild wire or puncture needle always slides along the edge of the “v” into the pericardial cavity. However, by using downward recanalization via jugular access, the guild wire or puncture needle always passes through the center point of the "v" shape, thus ensuring the correct direction and route of IVC cannulation. At this point, it is also feasible to insert a balloon catheter or intravascular stent via a jugular vein. However, in order to operate conveniently and further improve the safety of the procedure, especially to prevent arrhythmias and hematoma formation, we recommend the preferred transfemoral vein approach to insert balloon catheters or intravascular stents. In order to accomplish the procedure via the transfemoral vein, the guide wire must be drawn through the transfemoral vein and the JFV route should be established.

Another purpose of JFV route establishment is to facilitate the cannulation of the balloon catheter into the occluded segment of IVC. Because the guide wire can be pulled through the two ends to make it taut, the balloon catheter is relatively easy to pass the occlusion section along the tensioned guide wire, especially for the patients with long segmental occlusion (more than 3 cm). It is important not to apply too much force during the procedure, so the guide wire dose not cut the right atrium upper and lower entrance. In addition, use of the JFV route can also shorten the X-ray fluoroscopy time for patients and operators.

Our study showed a technical success rate of 97.1%. The 5-year secondary patency rate and survival rate was 96.8% and 90.0%, respectively. The complication rate was 8.8%, and no perioperative deaths were observed. Adverse event like IVC perforation, bleeding from IVC are serious in nature. Once IVC perforation occurs, the ruptured segment of IVC should be temporarily blocked with a dilated balloon to prevent massive abdominal bleeding. For patient with smaller rupture of IVC, the contrast agent may no longer overflow after the withdrawal of the balloon after 15–30 min due to the lower pressure of blood flood, and close observation can be made. Otherwise, covered vascular stent placement or surgical repair should be performed. In this study, one patient showed contrast agent outflow in the dilated segment after balloon angioplasty. A 26-mm diameter balloon catheter was introduced and dilated immediately to block bleeding, and no contrast agent was exuded after 15 min. The other patient showed rupture and bleeding of IVC, and surgical repair was performed after failure of balloon blockage.

Additionally, varies strategies can be used to improve the clinical efficacy of IVC recanalization, including catheter-direct thrombolysis, and use of a permanent stent or recoverable stent. Catheter-direct thrombolysis can be used for BCS patients with a fresh thrombus in the IVC^16,17^. A recoverable stent should be used for patients with massive IVC thrombus in order to avoid pulmonary thrombosis embolism^16,17,24,25^. For patients with recurrent BCS, a permanent stent can be chosen^17^.

In conclusion, the purpose and significance of JFV establishment is to facilitate the treatment of patients with long segmental occlusion, especially those with transfemoral access failure. It is a simple, safe and practical method.

## Data Availability

No datasets were generated or analyzed during the current study.
